# Management of Neglected Multiple Ligament Rupture of the Knee: A Case Report

**DOI:** 10.5704/MOJ.2111.018

**Published:** 2021-11

**Authors:** FA Maodah, S Rhatomy

**Affiliations:** 1 Department of Orthopedics and Traumatology, University of Airlangga, Surabaya, Indonesia; 2 Department of Orthopedics and Traumatology, Dr. Soeradji Tirtonegoro General Hospital, Klaten, Indonesia; 3 Faculty of Medicine Public Health Nursing, Universitas Gadjah Mada, Yogyakarta, Indonesia

**Keywords:** management, delayed or neglected injury, multiple ligament rupture

## Abstract

Delayed or neglected multiple ligament injury of the knee is challenging or doctors and physiotherapists. We report on a 36-year-old army man who presented to the outpatient orthopaedic department with complaints of pain and deformity of his right knee and an inability to weight-bear on the right leg. The examination of the right knee revealed an unreduced posterior dislocation of the knee with instability in all planes and a limited range of motion (ROM) from 10° to 100°. Quadriceps wasting was noted. Roentgenograms revealed a posterior dislocation of the knee. Magnetic resonance imaging (MRI) showed complete rupture of the anterior cruciate ligament (ACL), posterior cruciate ligament (PCL), medial collateral ligament (MCL) and the patellar tendon. A two-stage surgery was planned, involving soft tissue distraction with external fixation to restore the knee joint, followed by multiple ligament reconstruction. Three months after surgery, the patient could walk normally with ROM between 0° to 120°.

## Introduction

Knee dislocation after high energy trauma poses a major challenge to patients and treating physicians. The definition of a multi-ligament knee injury is commonly recognised as a tear of at least two of the four major knee ligament structures: the anterior cruciate ligament (ACL), the posterior cruciate ligament (PCL), the posteromedial corner (PMC) and the posterolateral corner (PLC)^1^.

The terms dislocation and multi-ligament knee injuries are often used interchangeably. As knee dislocations often result in multi-ligament knee injuries, a knee dislocation is typically characterised by rupture of both cruciate ligaments, with or without an associated grade III medial or lateral-sided injury. However, knee dislocations with one of the cruciate ligaments intact have been reported^1,2^.

For patients with no associated popliteal artery damage, the timing of surgical reconstruction also remains controversial. Early surgical intervention is advocated by many authors, with a general recommendation that reconstruction should be performed within the first three weeks. Proponents of early surgical reconstruction have reported better functional and clinical outcomes and have suggested that the risk of further chondral and meniscal injuries is reduced. However, many authors reported that early surgery resulted in stiffness, arthrofibrosis, and a reduced rate of return to work. In contrast, delayed surgery allowed for restoration of preoperative knee range of motion, recovery of soft tissues with a resolution of swelling, and possibly less stiffness and wound complications post-operatively^1-3^.

The cases of multiple ligaments knee injury in many of the studies were fresh cases, with acute admissions or directly to the emergency room. However, most patients came late to the hospital in developing countries, as they would consult a bonesetter first. There are only a few studies that reported on the management of these delayed or neglected multiple ligament rupture of the knee. In this study, we report the management of a neglected multiple ligament rupture of the knee.

## Case Report

A 36-year-old army man presented to the outpatient orthopaedic department with complaints of pain and deformity of his right knee and an inability to bear weight on the right leg. He had been involved in a traffic accident six months prior to presenting at the hospital. He had undergone some initial treatment in oil massage and splinting from the traditional indigenous bone setters.

**Fig 1:**
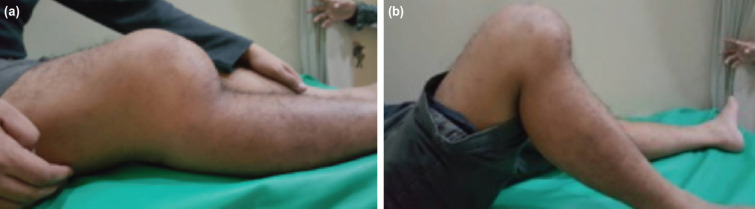
Clinical presentation, (a) maximal extension, (b) maximal flexion.

On examination, there was no tenderness in the right knee. There was an unreduced posterior dislocation of the knee with instability in all planes and a limited range of motion (ROM) from 10° to 100°. There was wasting of the quadriceps (Fig. 1). Clinical examination of the knee was negative for drawer, Lachman and varus tests and positive for the valgus test. Roentgenograms showed a posterior dislocation of the knee.

**Fig 2:**
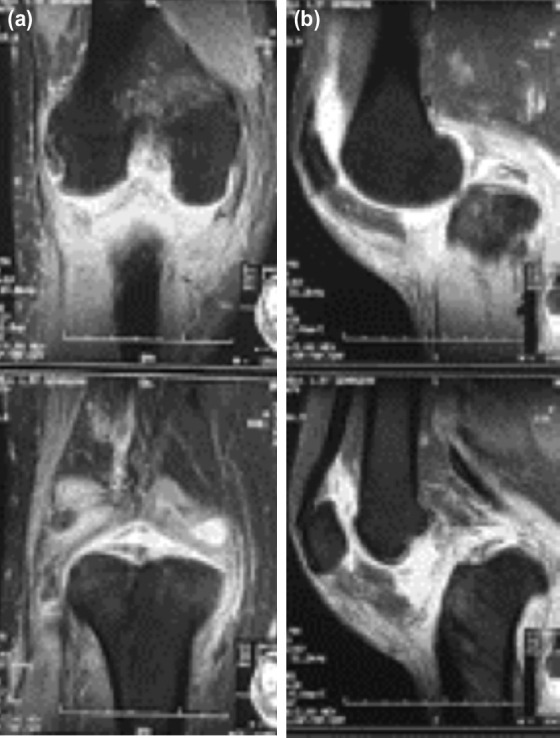
Magnetic resonance imaging of knee, (a) coronal view, (b) sagittal view.

From the magnetic resonance imaging (MRI), we found complete rupture of the anterior cruciate ligament (ACL), posterior cruciate ligament (PCL), medial collateral ligament (MCL) and the patellar tendon (Fig. 2). Therefore, we planned to perform a two-stage surgery, with the first stage of soft tissue distraction and the second stage of multiple ligament reconstruction.

In the first stage, we performed the soft tissue distraction. Under anaesthesia, we evaluated the ROM, which was 10° to 100°. Then we put one planar distraction for external fixation at the femoral and tibial bones.

In the ward, the patient underwent soft-tissue distraction 1mm/day, and after 30 days, the dislocation of the knee was resolved. We evaluated serial neurovascular examination while performing the distraction. There was no neurovascular injury right through to the final distraction.

For the second stage of the surgery, the multiple ligament reconstruction, we performed a medial parapatellar approach for knee arthrotomy and excised all the fibrotic tissues. The anterior cruciate ligament (ACL) was missing, and there was a complete rupture of the posterior cruciate ligament (PCL), medial collateral ligament (MCL) and patellar tendon (Fig. 2). We performed an open ACL and PCL reconstruction simultaneously, using hamstrings from the ipsilateral and contralateral leg; and the MCL and the patellar tendons were repaired with a simple suture.

The knee was kept in extension with a functional brace for one month, and then gradually, the flexion was increased, followed by non-weight bearing for another month. The patient was then allowed to bear partial weight until full weight-bearing gradually and was trained to strengthen the quadriceps muscles.

**Fig 3:**
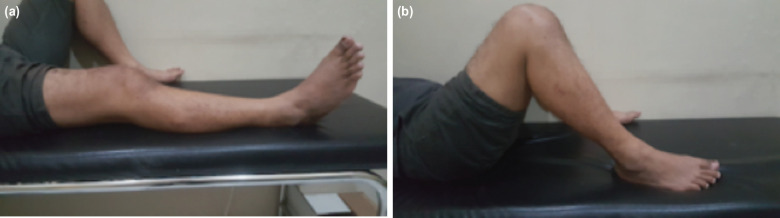
Clinical examination after surgery, (a) maximal extension, (b) maximal flexion.

Three months after surgery, the patient could walk normally with ROM between 0° to 120° (Fig. 3). In addition, there was an increased functional score of the knee, according to the Kujala and Tegner Lysholm Score Table I.

## Discussion

Delayed or neglected multiple ligament injury of the knee is very rarely reported in published studies. The controversy and discussion in the studies are usually related to the optimal treatment of these complex injuries in an acute setting^1^. However, in developing countries, patients often come late to a medical facility because they often go to a bonesetter first. As a result, it is a challenge for doctors and physiotherapists to manage this injury.

We found two studies that reported neglected knee dislocation, and both were cases performed with open knee surgery. One case was done with arthrodesis^3^, and the other case maintained the knee by placing crossed Steinmann pins across the joint with the knee held in 5° flexion^2^.

In our case, the patient is a young active army recruit, so we planned to preserve the knee joint. There are some reports of methods to manage delayed knee dislocation with contracture. Initial surgical management included soft tissue releases and lengthening of the hamstrings and the posterior capsule of the knee joint^4^. However, it is often difficult to obtain adequate knee extension with this procedure. Another method is serial extension casting, with and without simultaneous soft tissue release, and this procedure may be effective in mild situations. However, complications include fracture, posterior knee subluxation, and peroneal nerve injury. One other method is skeletal traction, and this procedure has been of benefit in severe cases but requires long-term hospitalisation^4,5^.

In our case, after discussion with the adult reconstruction team and the patient, we planned a two-stage surgery involving soft tissue distraction with external fixation to restore the knee joint, then followed with multiple ligament reconstruction. In the first stage of surgery, the patient underwent soft tissue distraction 1mm/day, and he was evaluated clinically and radiographically every week. After post-operative day 30, the knee joint completely regained its proper position, so we proceeded with the second stage of surgery.

In the second stage, we chose open surgery rather than arthroscopy to restore the ROM of the knee. We argued that open surgery is more optimal for the excision of all the fibrous tissue in cases with a delayed or neglected presentation, and for the soft tissue release. This is in accordance with previous studies which also performed open surgery for neglected cases^2,3^. Another difficulty is the unavailability of allografts in our country, so we have to use all available autografts for ligament reconstruction.

The final stage, which is the most important, is the rehabilitation program. This program must be evaluated continuously to strengthen the muscles, prevent stiffness and restore knee ROM.

The keys for successful treatment consist of a solid multidisciplinary team, adequate treatment options, intensive rehabilitation programs and post-operative pain management, in addition to patient compliance and a strong willingness to return to the normal function.
